# Acute cecal volvulus and large bowel obstruction in a patient with persistent Ladd bands and chronic malrotation: case report and review of literature

**DOI:** 10.1093/jscr/rjag143

**Published:** 2026-03-12

**Authors:** Neha Aftab, Enrique Romero-Vargas, Sean Lewis, Ambreen Sattar, Gul R Sachwani-Daswani, Kristoffer Wong

**Affiliations:** Department of Trauma and Acute Care Surgery, Hurley Medical Center, 1 Hurley Plaza, Flint, MI 48503, United States; Department of Surgery, Michigan State University, 200 East 1st street, 2nd floor, Flint, MI 48502, United States; Department of Trauma and Acute Care Surgery, Hurley Medical Center, 1 Hurley Plaza, Flint, MI 48503, United States; Department of Surgery, Michigan State University, 200 East 1st street, 2nd floor, Flint, MI 48502, United States; Department of Trauma and Acute Care Surgery, Hurley Medical Center, 1 Hurley Plaza, Flint, MI 48503, United States; Department of Surgery, Michigan State University, 200 East 1st street, 2nd floor, Flint, MI 48502, United States; Department of Radiology, Hurley Medical Center, 1 Hurley Plaza, Flint, MI 48503, United States; Department of Trauma and Acute Care Surgery, Hurley Medical Center, 1 Hurley Plaza, Flint, MI 48503, United States; Department of Surgery, Michigan State University, 200 East 1st street, 2nd floor, Flint, MI 48502, United States; Department of Trauma and Acute Care Surgery, Hurley Medical Center, 1 Hurley Plaza, Flint, MI 48503, United States; Department of Surgery, Michigan State University, 200 East 1st street, 2nd floor, Flint, MI 48502, United States

**Keywords:** bowel obstruction, Ladd bands, chronic malrotation, acute care surgery

## Abstract

Cecal volvulus occurs when the ascending colon and terminal ileum twist around the mesenteric pedicle, compromising bowel perfusion and leading to obstruction. This condition may result from congenital or acquired abnormalities affecting cecal fixation. Intestinal malrotation is rare in adults and is usually asymptomatic. We report the case of a 50-year-old male who presented with a large bowel obstruction due to cecal volvulus associated with chronic malrotation and Ladd bands. He underwent successful surgical treatment and was discharged home. This report discusses the atypical anatomy, clinical presentation, and diagnostic challenges of such cases.

## Introduction

Intestinal obstructions are among the most common surgical emergencies seen in clinical practice [1, 2]. Cecal volvulus is a rare, life-threatening cause of large bowel obstruction (LBO). It occurs when the cecum, ascending colon, and terminal ileum twist around the mesenteric pedicle, resulting in obstruction and potential compromise of bowel perfusion [2–5]. This condition typically develops in patients with congenital or acquired abnormalities of cecal fixation, which allow increased right colon mobility [1, 5–7]. Intestinal malrotation is a congenital anomaly caused by incomplete rotation of the midgut during embryologic development [2, 3, 5, 6, 8, 9]. It is often diagnosed in infancy, with an incidence of 1 in 500 live births [2, 8, 10].

Adult presentation is uncommon, occurring in about 0.2% of the population, and is often discovered incidentally or during evaluation for other causes of abdominal pain like gastritis or gastroesophageal reflux diseases or due to obstruction [4, 6, 8, 11, 12]. The causes of adult presentation are diverse. Many adults remain asymptomatic, while others present with symptoms such as intermittent or severe abdominal pain, chronic diarrhea, oral intolerance, malabsorption, hematemesis, hematochezia, or hemodynamic instability [8].

**Figure 1:**
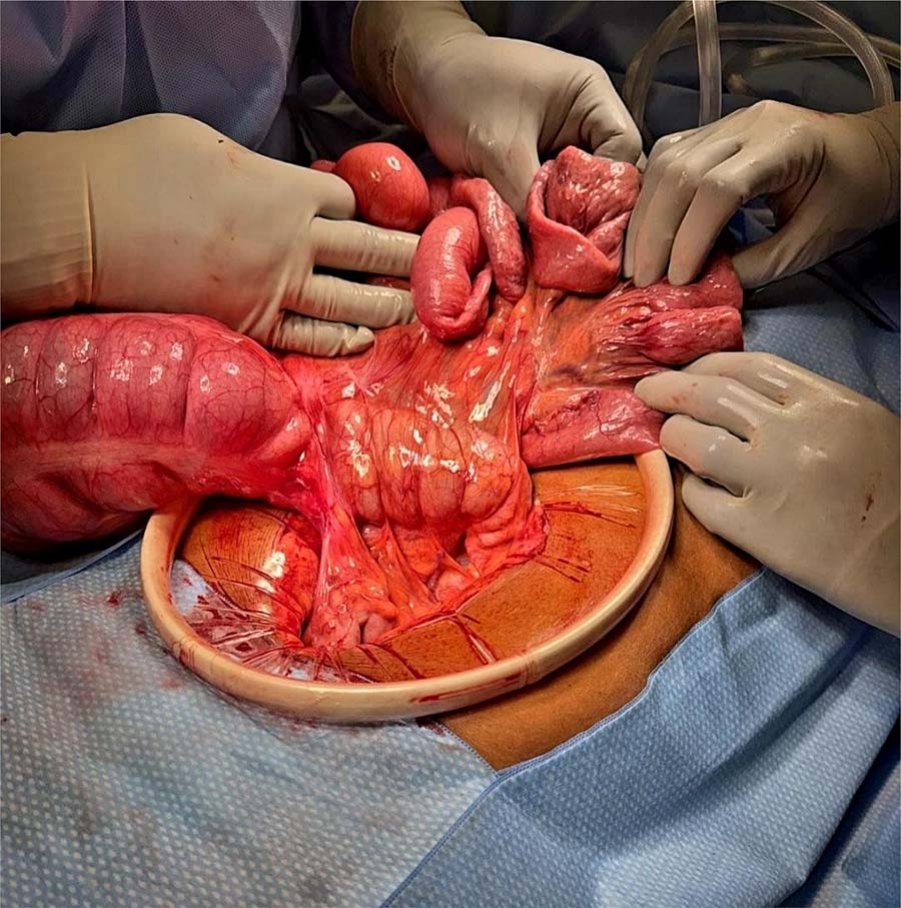
Intraoperative image demonstrating Ladd’s bands resulting in a cecal volvulus with dilated proximal small bowel and twisted cecum prior to ileocecectomy. Note the orientation of the small bowel anterior to the colon.

Diagnosing cecal volvulus or malrotation in adults can be challenging due to nonspecific clinical symptoms and its rarity. Computed tomography (CT) imaging is the preferred diagnostic tool, allowing confirmation of volvulus and identification of associated anatomic abnormalities [1]. Intestinal rotational issues may arise due to congenital abnormalities, as is the case with Ladd’s bands [1–3, 7]. These fibrous bands of peritoneal tissue can cause the cecum to be in an abnormal position, leading to complete obstruction presenting in newborns or mild obstructions that are often asymptomatic [2, 3, 7, 8, 10]. Mild obstructions may delay presentation in older patients [4, 8, 12]. Treatment is surgical (Fig. 1) and includes detorsion of the volvulus, division of any Ladd’s bands, and correction or fixation of the bowel to prevent recurrence [7]. We present a case of a 50-year-old male with acute cecal volvulus and persistent congenital intestinal malrotation with Ladd’s bands.

## Case presentation

A 50-year-old male with hypertension and cannabis use disorder presented to the emergency department with 36 h of worsening, diffuse abdominal pain without radiation. He also reported nausea, non-bloody vomiting, and constipation. Imaging revealed mildly to moderately dilated small and large bowel loops, with significant enlargement of a colon loop in the lower abdomen, indicating possible bowel obstruction and ileus. He underwent an exploratory laparotomy the same day. Intraoperatively, the cecum was found unattached to the right lateral abdominal wall and instead adhered to the distal small bowel mesentery by a thick, white peritonealized band. The cecum was torsed. The peritoneal bands were sharply lysed, and the cecum was manually detorsed.

The entire small bowel mesentery was anterior to the transverse colon, resulting in an anterior configuration of the duodenum and head of the pancreas with respect to the transverse colon. Due to concerns for cecal viability, a blue load gastrointestinal anastomosis (GIA) stapler was used to exclude the compromised segment of cecum, effectively performing an ileocecectomy with side-to-side functional end-to-end ileocolostomy. The patient recovered excellently postoperatively with immediate improvement in his abdominal discomfort and nausea. He had robust return of bowel function by postoperative day-3, was tolerating a regular diet by day-4, and was accordingly discharged home on this day with no complications at the time of writing this manuscript, about 2-months after his presentation.

## Discussion

**Figure 2:**
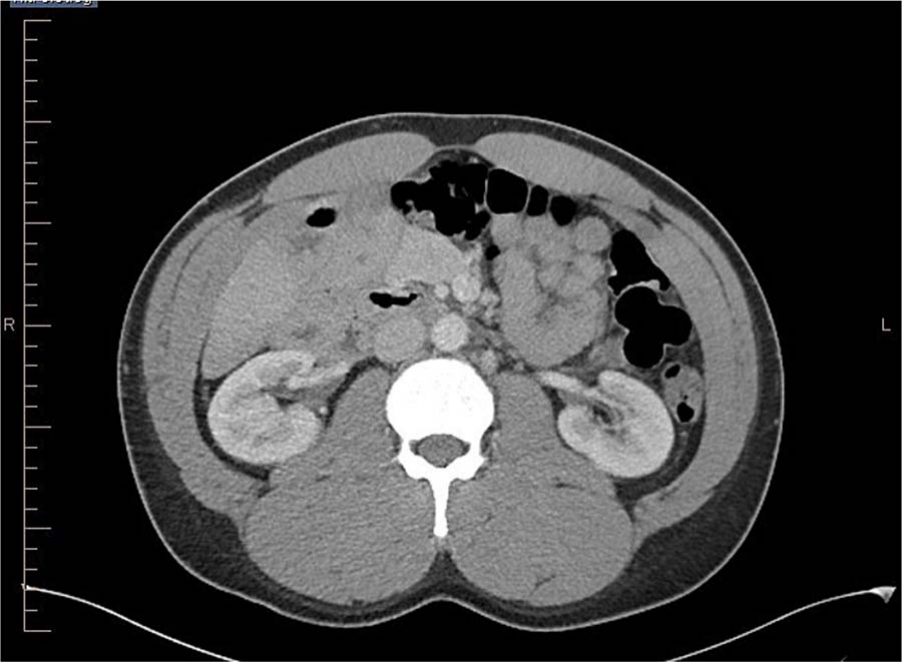
Axial view of abdominal CT scan with contrast showing the reversal of SMA and SMV.

Around week 6 of fetal development, the midgut loop herniates into the umbilical cord. It then rotates 270° counterclockwise around the superior mesenteric artery, with the first 90° occurring during herniation and the remaining 180° as it returns to the abdominal cavity. By week 12, the midgut re-enters the abdomen, placing the cecum and colon on the right and the jejunum and ileum on the left. Malrotation arises when the normal 270° counterclockwise rotation fails to occur [13] (Fig. 2). In such cases, the cecum may remain high and mobile, often located in the right upper quadrant, rather than fixed in the right lower quadrant. Ladd bands, which are abnormal fibrous peritoneal bands, develop when the intestines fail to rotate properly. These bands extend from the cecum across the duodenum, abnormally anchoring the bowel to the retroperitoneum. The presence of a hypermobile cecum and Ladd bands can result in twisting of the cecum around the mesenteric base, leading to cecal volvulus and LBO. This condition most commonly presents in childhood, with a peak incidence in the first few months of life; 75%–85% of cases present within the first month of life [8].

Symptomatic malrotation in adults is rare, with an incidence of 0.2%. Incidental diagnosis often occurs during imaging or surgery for unrelated conditions. The literature describes two distinct patterns of adult midgut malrotation: acute and chronic. The chronic form, which is more prevalent, typically presents with crampy abdominal pain and diarrhea. Diagnostic delays are common due to nonspecific symptoms resulting from bowel compression by Ladd bands. The acute form, as observed in this case, presents with signs and symptoms of acute bowel obstruction, with volvulus being the most frequent cause of obstruction in adults with malrotation. Cecal bascule, an uncommon variant of cecal volvulus, is characterized by anterior and upward folding of the cecum. In contrast, this patient exhibited a classic cecal volvulus with mesenteric torsion [14].

**Figure 3:**
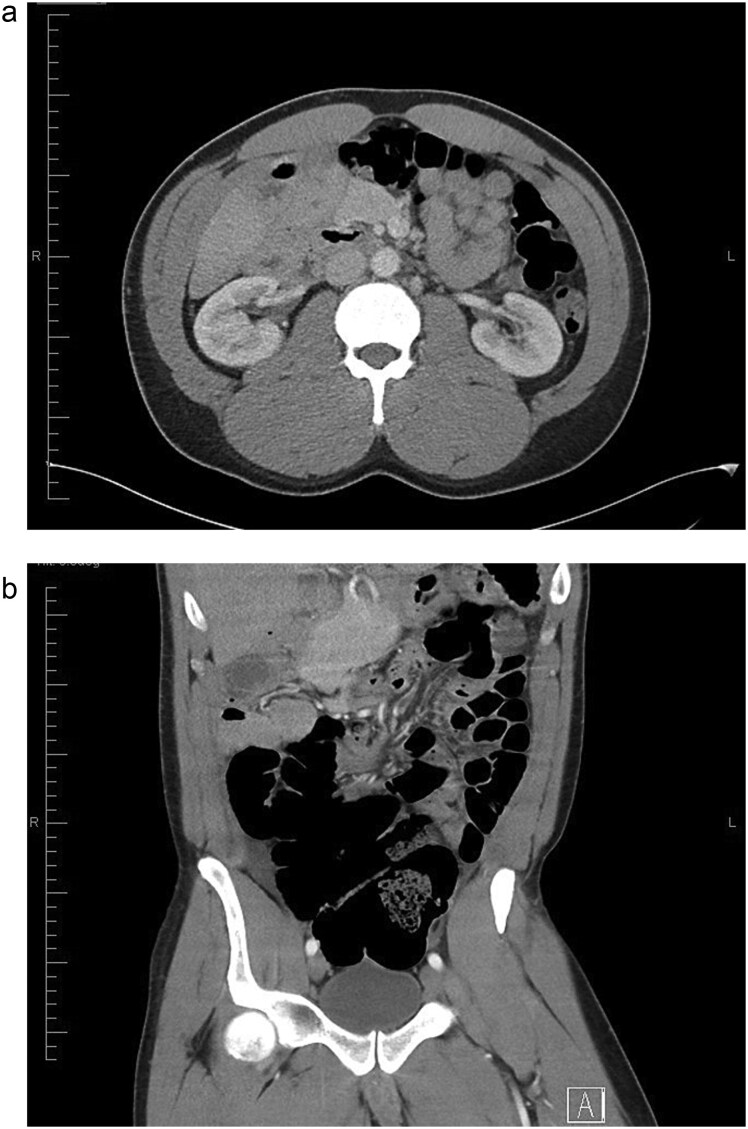
(a) Axial view of CT abdomen with contrast showing swirl sign i.e. swirling of bowel and mesentery; suggesting intestinal volvulus (superior to SMA and SMV. (b) Coronal view of CT abdomen with contrast showing swirl sign i.e. swirling of bowel and mesentery; suggesting intestinal volvulus.

**Figure 4:**
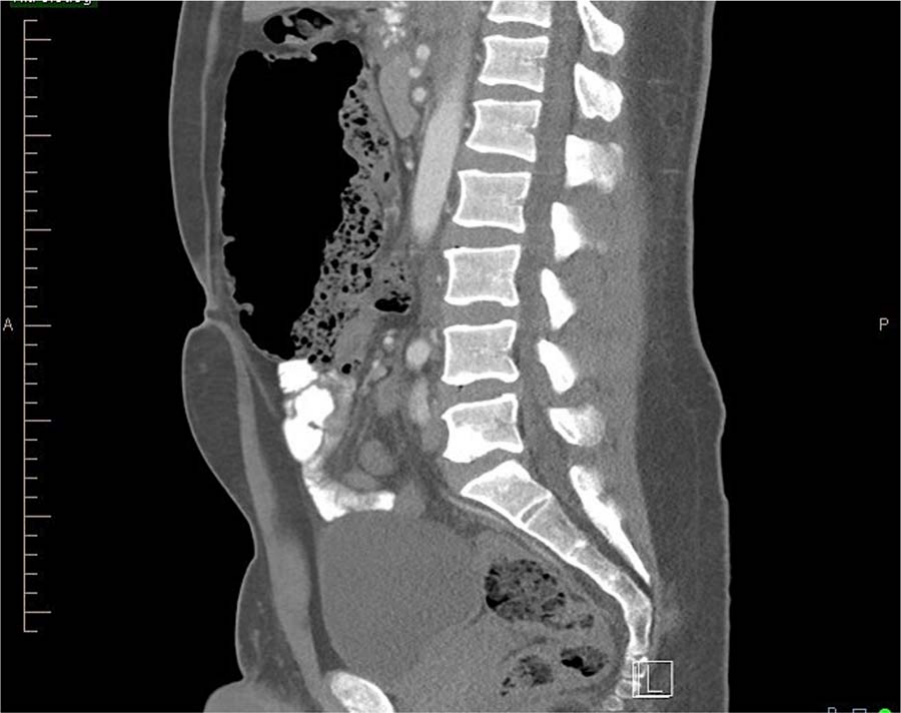
Sagittal view of CT abdomen showing higher and more anterior location of cecum; suggesting a hypermobile cecum (Normally the cecum is located lower in the right lower quadrant).

The gold standard for diagnosing malrotation is an upper gastrointestinal (GI) series or a CT scan with oral and intravenous (IV) contrast. In adults, CT is preferred. Characteristic imaging findings include the ‘whirlpool’ sign (Fig. 3a and b), the superior mesenteric vein (SMV) rotation sign, where the SMV lies anterior to the superior mesenteric artery (SMA), as well as right-sided positioning of the bowel, duodenojejunal flexure, and cecum [8] (Fig. 4).

The original Ladd procedure, first described by William Ladd for pediatric patients, involves three main steps to treat malrotation and volvulus: detorsing the volvulus, relieving extrinsic compression of the cecum by the duodenum, and separating the duodenum and colon to free the cecum from the posterior abdominal wall, followed by repositioning the intestine to reduce the risk of recurrent torsion [7]. This approach was later adapted for adults. The laparoscopic technique, introduced in the early 2000s, is preferred for stable patients, though outcomes and 30-day mortality rates are similar for both open and laparoscopic approaches [15].
